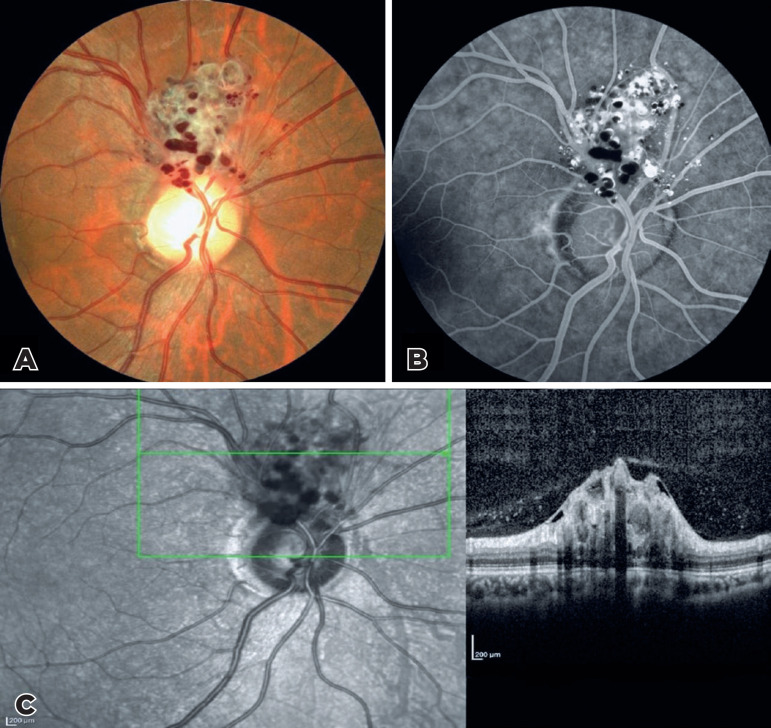# Multimodal imaging characteristics of peripapillary cavernous hemangioma

**DOI:** 10.5935/0004-2749.2024-0253

**Published:** 2024-12-18

**Authors:** Kemal Tekin, Mehmet Yasin Teke

**Affiliations:** 1 Ophthalmology Department, Ulucanlar Eye Training and Research Hospital, Ankara, Turkey

A 49-year-old female patient was referred to our re-tina clinic because of a peripapillary vascular tumor detected during a routine eye examination. The fundus examination showed peripapillary grape-like clusters of dilated sac-like aneurysms filled with dark red blood (Figure 1A). Fundus fluorescein angiography revealed typical blood levels. There was plasma-erythrocytic separation was observed within some aneurysms because of pooling of the dye in the superior plasma (producing hyperfluorescence) and inferior sedimented red blood cells (producing hypofluorescence) (Figure 1B). Spectral--domain optical coherence tomography passing through the lesion showed grape-like bunches of hyporeflective vesicular formations surrounded by a ring with a hyperreflective edge involving the inner retinal layers (Figure 1C).

Cavernous hemangiomas of the retina may follow the courses of major veins or manifest in the peripapillary area with aneurysmal venules dilation. They may co-occur with cutaneous or central nervous system hemangiomas^(^1^)^. They can be diagnosed using imaging results in combination with the distinctive appearance of the fundus^(^1^)^

**Figure:**